# 105 Physical activity in relation to WHO guidelines, among Swedish adults during the COVID-19 pandemic. A repeated cross-sectional study

**DOI:** 10.1093/eurpub/ckae114.096

**Published:** 2024-09-26

**Authors:** Bodhini Samaratunga, Birgitta Kerstis, Daniel Lindberg, Maria Elvén, Charlotta Hellström, Jonas Stier, Susanna Lehtinen-Jacks

**Affiliations:** Mälardalen University, Västerås, Sweden; Mälardalen University, Västerås, Sweden; Örebro University, Örebro, Sweden; Mälardalen University, Västerås, Sweden; Mälardalen University, Västerås, Sweden; Mälardalen University, Västerås, Sweden; Mälardalen University, Västerås, Sweden

## Abstract

**Purpose:**

Assess changes in health-promoting physical activity (HPPA), according to the WHO guidelines, among Swedish adults during the COVID-19 pandemic. HPPA has been ignored as an outcome in the physical activity (PA) studies during the pandemic.

**Methods:**

A population-based repeated cross-sectional study was conducted among 18-79-year-old Swedes (n = 1035 in December 2020 [response rate 52%], n = 1095 in January 2022 [55%], n = 1027 in December 2022 [55%]). Data was collected via online questionnaires, including sex, age, civil status, education, employment, and country of birth. PA was measured using the International Physical Activity Questionnaire-Short Form (IPAQ-SF). In 2020, recall questions about PA one year earlier were included. The WHO guidelines for moderate, vigorous, or combined HPPA were applied, and the proportions of respondents completing the guidelines for these three HPPA categories were calculated.

Differences in proportions of moderate, vigorous, and combined HPPA across the survey timepoints were tested by chi-square tests, and by McNemar tests concerning differences between the recall in 2019 and December 2020. Binary logistic regression analyses were performed for women and men to assess whether the survey timepoints or the sociodemographic variables were independently associated with HPPA. Interaction terms between each sociodemographic factor and survey timepoint were added to the models to assess differences in changes in HPPA in different sociodemographic subgroups.

**Results:**

While the proportions of moderate, vigorous, and combined HPPA decreased substantially from 2019 (recall) to 2020 in both sexes, the changes seen between 2020 and 2022 were statistically non-significant. No significant interactions between sociodemographic factors and survey timepoint were observed, and survey timepoint was not associated with HPPA in the logistic regression models. Factors most consistently associated with the different HPPA categories were education in women and employment in men. In women, compulsory and/or high school indicated less HPPA than university education. Among men, white-collar and/or retired had less HPPA than the blue-collar employment category.

**Conclusions:**

No clear changes in HPPA were observed between December 2020 and December 2022. Differences in HPPA according to education and employment indicate need for research on targeted PA promotion measures.

**Support:**

Mälardalen University, Sweden.

